# Multichannel Inductive Sensor Based on Phase Division Multiplexing for Wear Debris Detection

**DOI:** 10.3390/mi10040246

**Published:** 2019-04-13

**Authors:** Sen Wu, Zhijian Liu, Haichao Yuan, Kezhen Yu, Yuefeng Gao, Liankun Liu, Xinxiang Pan

**Affiliations:** 1 College of Marine Engineering, Dalian Maritime University, Dalian 116026, China; dlmuwusen@163.com (S.W.); liuzhijian@dlmu.edu.cn (Z.L.); yuanhc@dlmu.edu.cn (H.Y.); yukezhen17@hotmail.com (K.Y.); gaoyuefeng@dlmu.edu.cn (Y.G.); laulienkun@163.com (L.L.); 2 College of Navigation, Guangdong Ocean University, Zhanjiang 524088, China

**Keywords:** inductive sensor, multichannel, phase division multiplexing, synchronized sampling, wear debris, microfluidics

## Abstract

Inductive wear debris sensor has been widely used in real time machine lubricant oil condition monitoring and fault forecasting. However, the small sensing zone, which is designed for high sensitivity, of the existing sensors leads to low throughput. In order to improve the throughput, a novel multichannel wear debris sensor that is based on phase division multiplexing is presented. By introducing the phase shift circuit into the system, multiple sensing coils could work at different initial phases. Multiple signals of sensing coils could be combined into one output without information loss. Synchronized sampling is used for data recording, and output signals of multiple sensing coils are extracted from the recorded data. A four-channel wear debris sensor system was designed to demonstrate our method. Subsequently, crosstalk analysis, pseudo-dynamic testing and dynamic testing were conducted to check the sensing system. Results show that signals of four sensing coils could be simultaneously detected and the detection limit for ferrous wear debris is 33 μm. Using the presented method, real time wear debris detection in multiple channels could be achieved without increasing the number of excitation source and data acquisition equipment.

## 1. Introduction

Wear debris contains considerable information regarding machine’s working status and lubricant condition. The detection of wear debris could track and detect machine failure in time. Many methods have been developed for real time wear debris detection, such as bulk measurement, capacitance, inductance, acoustic, and optical/imaging detection [1,2,3,4,5]. Among these methods [6,7,8,9,10], an inductive sensor is proved to be the most practical method for the following reasons. Inductance detection could differentiate between ferrous and nonferrous debris [11,12], and it is insensitive to air bubbles and water droplets [13,14]. Some inductive sensors, such as MetalSCAN (an on-line oil debris sensor made by GasTOPs Ltd. ), have been utilized in the wind turbine gearbox and aviation machinery [15,16,17,18]. Similar to other sensors, the sensitivity is an important feature for the inductive sensor. In order to increase the sensitivity, a two-dimensional (2D) planar sensor coil or three-dimensional (3D) solenoid with a small sensing zone have been developed [19,20,21,22,23,24]. In addition, ferrite cores and dual excitation sources have also been used to concentrate the magnetic lines and to further improve the sensitivity [25,26,27]. Unfortunately, the small sensing zone of the existing sensor would lead to low throughput. It is fact that enlarging the cross-sectional area of the sensor or increasing the flow velocity can improve the throughput. However, the sensitivity would be decreased at the same time. Accordingly, improving the throughput without decreasing the sensitivity for inductive sensor is a great challenge.

The concept of multichannel wear debris sensor was proposed to improve the throughput with existing sensors. Du et al. presented a parallel detection multichannel oil wear debris sensor [28]. The sensing system requires seven sampling channels to monitor seven sensing coils. Hence, the system would be complex and expensive. A resonant frequency division multiplexing wear debris sensor was presented with only one set of input signal and output signal to monitor multiple sensors with only one sampling channel [29,30]. However, each of the sensing channels/coils is connected in parallel with an external capacitance to have different resonant frequencies, which may lead to different sensitivities. Zhu et al. proposed a 3 × 3 wear debris sensor array for lubricant oil condition monitoring that is based on time division multiplexing [31]. Each of the sensors is sequentially activated in different timeslots by two multiplexers. However, a certain settling time (~5 μs) in each timeslot is needed to remove the glitch noise and the transient response, which leads to much of the recorded data being unavailable.

To overcome the above mentioned limitations, a novel multichannel wear debris sensor based on phase division multiplexing is presented. Signals of multiple sensing channels/coils could be simultaneously detected with only one sinusoidal excitation signal and one sampling channel. Multiple sensing coils in the sensing system work at different initial phases. The sine waves from multiple sensing coils are combined into one output signal. The synchronized sampling method is used to record the only one output signal, and then the signals of multiple sensing coils could be extracted from the sampled data. With the presented method, high throughput could be obtained without increasing the number of the excitation source and the data acquisition equipment (DAQ).

## 2. System Design

### 2.1. Sensing Mechanism

**Figure 1:**
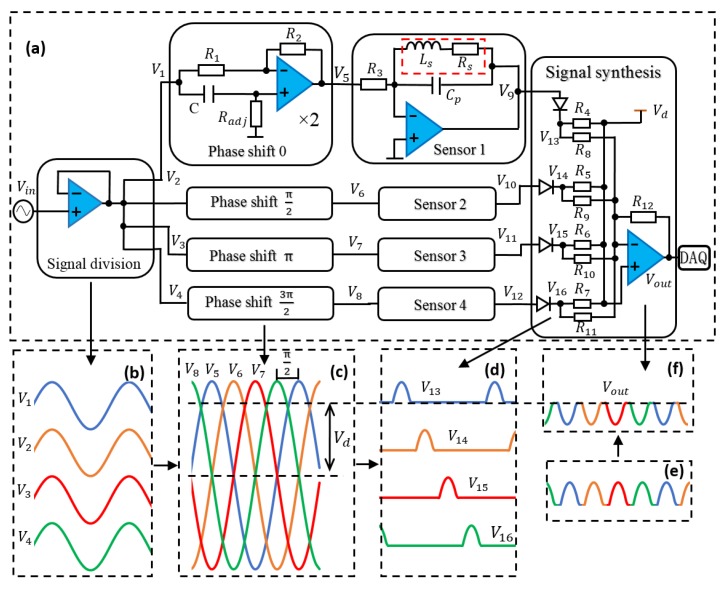
Illustration of sensing mechanism, (**a**) Design of multichannel sensor circuits, $R_{1}$ = $R_{2}$ = 10 kΩ, C = 100 pF, $R_{\mathrm{adj}}$ (3296X-1-502LF, Bourns Inc., Columbia, CA, USA) ranges from 0 to 5 KΩ, $\;R_{3}$ = 100 Ω, $R_{4}$ = $R_{5}$ = $R_{6}$ = $R_{7}$ = 200 Ω, $R_{8}$ = $R_{9}$ = $R_{10}$ = $R_{11}$ = $R_{12}$ = 6 kΩ, amplifier (AD8045, Analog Device Inc., Norwood, MA, USA), (**b**) Four signals that were divided from sinusoidal excitation signal, (**c**) Signals at sensor unit output port with $\frac{\pi }{2}$ phase difference, (**d**) Signals at output port of diodes, (**e**) Integrated signal of $V_{13}$, $V_{14}$, $V_{15}$, $V_{16}$, and (**f**) output signal $V_{\mathrm{out}}$.

To demonstrate the sensing mechanism, we present the solution for four channels here. Using a similar solution, a larger number of channels could also be installed in the sensing system. Figure 1 shows the phase division multiplexing sensing mechanism for wear debris detection. The wear debris information of four channels/coils could be obtained by only one set of detection electronics. The sensing system consists of signal division, phase shift, sensor, and signal synthesis units (Figure 1a). Signal division unit is used to divide the input sinusoidal excitation signal $V_{in}$ into four branches (Figure 1b). The phase shift unit could shift the four signal branches to have a phase difference ($P_{d}$) of $\frac{\pi }{2}$ between adjacent signals (Figure 1c). The sensor unit is used for sensing the wear debris signal with the resonant circuit [32]. The signal synthesis unit cut out the peak waveform of the four signals from sensor units (Figure 1d), and then the signals ($V_{13}$, $V_{14}$, $V_{15}$, $V_{16}$) are integrated into one signal with the four signals’ peak values being reserved (Figure 1e). The polarity of the integrated signal (Figure 1e) would be inverted to $V_{\mathrm{out}}$ (Figure 1f) after it passes through the gain inverting amplifier in the signal synthesis unit. The output signal $V_{\mathrm{out}}$ is sampled by DAQ while using the synchronized sampling method.

The principle of four units in the sensing system is shown in Figure 1a. Signal division unit is a voltage follower to eliminate the unstable input signal that is induced by the waveform generator’s internal resistance. Each sensing coil could be modelled as an inductance $L_{s}$ in series with a resistance $R_{s}$, shown in red dotted frame in sensor unit (Figure 1a) [32]. Each micro 3D solenoid is connected to an external capacitor $C_{p}$ in parallel to form a parallel resonant circuit. The signal synthesis unit contains four diodes (1N60P, Semtech Electronics Ltd., Shatin, Hongkong) and one summing circuit with a high level voltage $V_{d}$ being provided (Figure 1a). The diode only allows a forward voltage to pass through. Without the reverse voltage passing through, the diode would only turn on when the signal is higher than $V_{d}$. With the existence of four diodes and $V_{d}$, signals higher than $V_{d}$ could be obtained. The summing circuit is used to integrate the four signals ($V_{13}$, $V_{14}$, $V_{15}$, $V_{16}$) into one signal (Figure 1e). DAQ is grounded to $V_{d}$ to record the output signal higher than $V_{d}$. From the output signal, the debris information of four sensing coils could be obtained.

The key for the sensing system is how to shift the four signal branches to have a phase difference ($P_{d}$) of $\frac{\pi }{2}$ between the adjacent signals. A typical phase shift circuit is used in our design (Figure 1a). The phase shift unit contains two phase shift circuits. For the single phase shift circuit, it contains two resistors $R_{1}$, $R_{2}$ ($R_{1}$ = $R_{2}$), one adjustable resistor $R_{\mathrm{adj}}$, and one capacitor *C*. The phase shift angle of single phase shift circuit is

(1) $$\theta =\tan^{-1}\frac{2}{R_{\mathrm{adj}}\omega C-\frac{1}{R_{\mathrm{adj}}\omega C}}$$

In Equation (1), ω is angular velocity of input signal. When $R_{\mathrm{adj}}\to +\infty$,

(2) $$\theta =\underset{R_{\mathrm{adj}}\omega C\to +\infty }{\lim}\tan^{-1}\frac{2}{R_{\mathrm{adj}}\omega C-\frac{1}{R_{\mathrm{adj}}\omega C}}=0{}^{\circ}$$

When $R_{\mathrm{adj}}\to \frac{1}{\omega C}$,

(3) $$\theta =\underset{R_{\mathrm{adj}}\omega C\to 1}{\lim}\tan^{-1}\frac{2}{R_{\mathrm{adj}}\omega C-\frac{1}{R_{\mathrm{adj}}\omega C}}=\frac{\pi }{2}$$

When $R_{\mathrm{adj}}\to 0^{+}$,

(4) $$\theta =\underset{R_{\mathrm{adj}}\omega C\to 0^{+}}{\lim}\tan^{-1}\frac{2}{R_{\mathrm{adj}}\omega C-\frac{1}{R_{\mathrm{adj}}\omega C}}=\pi$$

Hence, the single phase shift circuit has a phase shift angle, ranging from 0 to π by adjusting the value of resistor $R_{\mathrm{adj}}$ from +∞ to $0^{+}$.

As each phase shift unit contains two phase shift circuits, the acquirable phase shift angle range is 0 to 2π. Four phase shift units are used to achieve the phase shift angle of 0/$\frac{\pi }{2}$/π/$\frac{3\pi }{2}$ for four channels separately, as shown in Figure 1a. In each phase shift unit, there are two capacitors C and two adjustable resistors $R_{\mathrm{adj}}\;1$ and $R_{\mathrm{adj}}\;2$. The values of C are the same in each unit, C = 100 pF. The two resistors in each unit are adjusted together to achieve the desired phase shift angle. According to Equations (1)–(4), Table 1 provides one solution in theory.

However, the actual values of $R_{\mathrm{adj}}$ are a little different from the values in theory. In order to obtain the accurate value of $R_{\mathrm{adj}}$, especially when the desired value is relatively small, an adjustable resistor with small value range is needed. For example, the value 1590 Ω that is required for phase shift of $\frac{\pi }{2}$ ($\frac{1}{\omega C}=\frac{1}{2\pi f\times C}≅1590\;\Omega$, f=1 MHz) could be accurately obtained by adjusting the 5 kΩ range adjustable resistor that was used in the experiment. As the resistance of $R_{\mathrm{adj}}$ used in the experiment cannot reach +∞, the phase shift angle of phase shift unit 1 is larger than 0 rad. Consequently, the phase angles of other three sensing coils’ signals should be further shifted, and the actual values of $R_{\mathrm{adj}}$ in the phase shift units are smaller than the value in Table 1.

### 2.2. Signal Extraction

**Figure 2:**
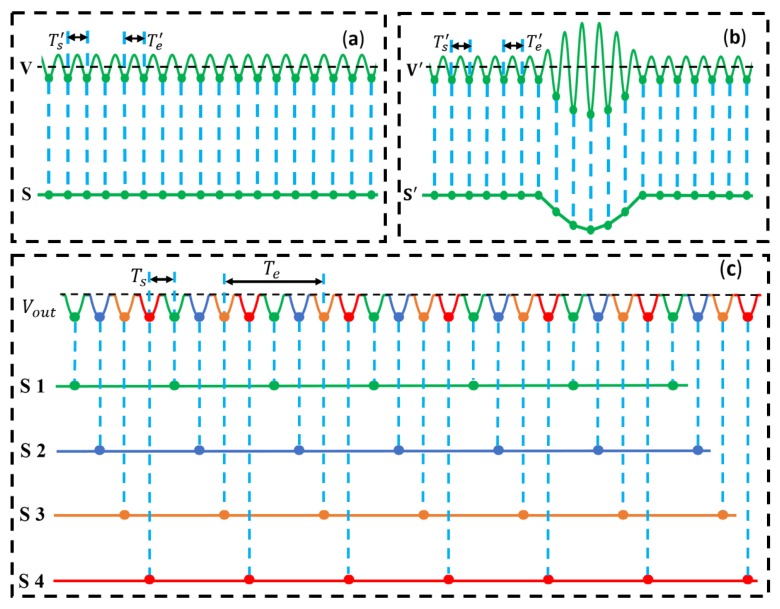
(**a**) Principle of synchronized sampling with no particle passing through sensing coil, $T_{e}^{\prime }$ is the period of sinusoidal excitation signal, $T_{s}^{\prime }$ is the period of sampling, $T_{e}^{\prime }=T_{s}^{\prime }$, **V** is the sine wave from single sensor, **S** is the voltage output of the sensing coil. (**b**) Synchronized sampling with particle passing through sensing coil, the amplitude of sine wave $V^{\prime }$ is enlarged, and the pulse appears in the voltage output $S^{\prime }$. (**c**) Synchronized sampling and signals extraction method for four-channel sensor, $T_{s}$ is the period of sampling, $T_{e}$ is the period of excitation signal, $4T_{s}=T_{e}$, signals S1, S2, S3, S4 are the voltage outputs of four sensing coils.

The output signal $V_{\mathrm{out}}$ is recorded by the synchronized sampling method. DAQ is synchronized with sinusoidal excitation source by using the same clock signal as the master time. For a single sine wave, Figure 2a,b shows the principle of synchronized sampling. By setting the period of data sampling $T_{s}^{\prime }$ equal to the period of sine wave $T_{e}^{\prime }$ and adjusting the initial phase of sampling clock to have $\frac{\pi }{2}$ phase difference with sine wave, the peak values of sine wave could be sampled. As shown in Figure 2a, the negative peak values of single sine wave V are recorded. By linking the peak values together, the voltage output S could be obtained. When no particle passes through the sensing coil, the voltage output S of the sensing coil is a straight line. When ferrous wear debris passes through the sensing coil, the peak values of sine wave $V^{\prime }$ are enlarged, as shown in Figure 2b. Due to the negative peak values being recorded, negative particle pulse induced by ferrous particle appears in the voltage output $S^{\prime }$. The amplitude of voltage pulse is corresponding with the wear debris size passing through the sensing coil [20].

For the multichannel sensor system, the output signal $V_{\mathrm{out}}$ could also be recorded by the synchronized sampling method [31,33]. Supposing that the number of channels is N, excitation frequency is $f_{e}$, sampling frequency is $f_{s}$, the period of sampling is $T_{s}$, and the period of excitation signal is $T_{e}$, $f_{s}=1/T_{s}$ and $f_{e}=1/T_{e}$. In order to record every peak values of output, $f_{e}$ and $f_{s}$ should satisfy the equation:

(5) $$f_{s}=N\times f_{e}$$

There are four channels in our experiment, so we only present, but are not limited to, the signal extraction method for four channels. In our experiment, the output signal of sensor system $V_{\mathrm{out}}$ (Figure 2c), same to $V_{\mathrm{out}}$ in Figure 1f, includes the peak values of four sine waves from four sensing coils. In order to obtain the signals of four sensing coils from the output signal $V_{\mathrm{out}}$, the synchronized sampling and the signal extraction methods for four-channel sensor are shown in Figure 2c. According to Equation (5), the frequency of data sampling ($f_{s}$) is set to be four times of the frequency of excitation signal ($f_{e}$). The peak values in the recorded data array sequentially represent the four coils’ output voltages, and they are divided into four subsets. Figure 2c shows the four coils’ peak value subsets (four rows of different color dots). The output signals of four sensing coils could be obtained by linking the peak values of each subset.

The final outputs (S1, S2, S3, S4 shown in Figure 2c) of four sensing coils are independent from each other. There are two meanings for “independent”: (1) When only one particle passes through one of the four sensing coils, only one pulse would appear in its output, no pulse would appear in other sensing coils’ outputs; (2) when two particles are passing through two sensing coils at the same time, there would be two pulses separately appearing in two outputs at the same time, and two pulses would be counted. If four outputs were not separately extracted from the collected output signal, only one pulse would be counted when multiple particles are passing through multiple sensing coils at the same time. Thus, with the presented signal extraction method, particles’ pulses of four sensing coils could be counted with no pulses overlapping among the sensing coils.

### 2.3. Measurement Setup

**Figure 3:**
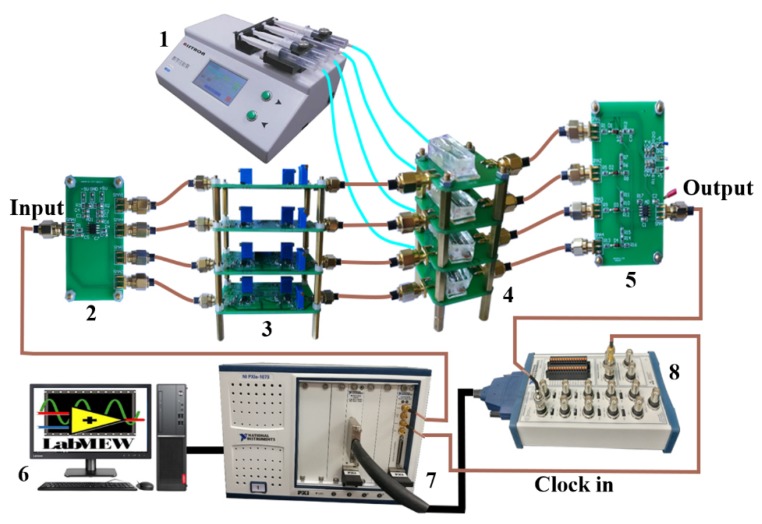
Measurement setup of multichannel sensing system with following components: 1. Micro-syringe pump, 2. Signal division unit, 3. Phase shift unit, 4. Sensor unit, 5. Signal synthesis unit, 6. Computer with LabVIEW software, version 7. Waveform generator PXI-5422 and data acquisition equipment PXIe-6124 multifunction I/O module, 8. Shielded block with Bayonet Nut Connector (BNC).

Figure 3 shows the measurement setup. A waveform generator (PXI-5422, National Instruments, Austin, TX, USA) was used to generate 1 MHz, 1.6 Vpp sinusoidal excitation signal ($V_{\mathrm{in}}$). The output signal $V_{\mathrm{out}}$ was recorded by data acquisition equipment (PXIe-6124, National Instruments, Austin, TX, USA) with a sampling rate of up to 4 MS/s (Megasamples per second). To perform the sensing mechanism for the four-channel wear debris sensor: (1) The sinusoidal excitation signal was connected to the input port; (2) The resonant frequency (1.05 MHz) of parallel resonant circuit was shifted to slightly higher than the excitation frequency (1 MHz) by adjusting capacitance $C_{p}$ in the sensor unit (Figure 1a). At this working frequency, ferrous and nonferrous metallic debris could be differentiated from the polarity of impedance response [30]; (3) By adjusting the phase shift unit, the initial phases of signals at the output port of sensor units were shifted to have a phase difference $P_{d}$ of $\frac{\pi }{2}$ between the adjacent signals. Subsequently, the peak waveforms of four signals were integrated into one output by a signal synthesis unit; (4) The peak values of output signal $V_{\mathrm{out}}$ were recorded by DAQ. DAQ was phase-locked with waveform generator by using the same master clock signal (200 MHz). By adjusting the phase difference between the output signal and the sampling clock signal, the peak values of output signal could be collected. During the data sampling procedure, the temperature change would lead to the value change of $R_{\mathrm{adj}}$. The changed $R_{\mathrm{adj}}$ would further change the initial phase of sensing coil’s output signal, which would lead to the DAQ missing the peak value of the output signal. Thus, a cooling fan is used to stabilize the value of resistor $R_{\mathrm{adj}}$ and other components.

Automatic hot air winder winded the sensing coils used in our experiment (YZE-1200, Dongguan YinZhuoEn Precision Automation Co., Ltd., Dongguan, China). 3D solenoid coils (390 μm in axial length, 400 μm in internal diameter, 1700 μm in external diameter, 65 μm in wire diameter) were fixed by PDMS (polydimethylsiloxane), with a fluidic channel in the center. Agilent E4980A precision LCR meter measured the inductances $L_{s}$ of four 3D solenoid coils. In order to ensure each sensing coil reaches at an identical resonant frequency, four corresponding capacitances $C_{p}$ were calculated, as listed in Table 2.

## 3. Experiment Results and Discussion

### 3.1. System Function Validation

**Figure 4:**
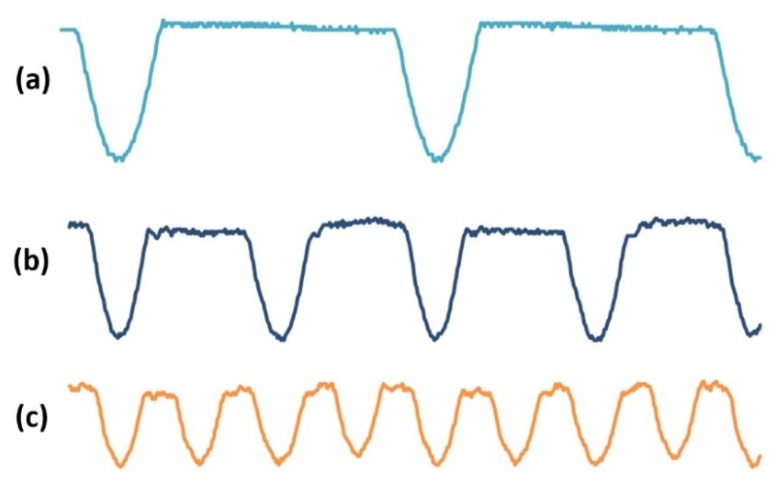
Signal synthesis results of signals from, (**a**) One channel, (**b**) Two channels with πphase difference, and (**c**) Four channels with $\frac{\pi }{2}$ phase difference.

To validate the function of the whole sensing system, the testing procedure was divided into three steps. An oscilloscope was used to monitor the output signal of each testing port. Firstly, each unit in the sensing system was separately tested to ensure that the function of single unit is achieved. Secondly, the units were connected into the sensing system one by one, as shown in Figure 1a, from left to right. The output signal of each unit needs to be checked after being connected to the former unit. In order to ensure that the synthesis unit could obtain the expected signal of each sensing coil, only one signal from sensor unit was connected to the input port of signal synthesis unit in each time. Figure 4a shows the output signal with only one sensing coil’s signal, connected to signal synthesis unit, which is the same with the signal in Figure 1d. Thus, the peak waveform was cut out from sine wave signal. Lastly, two signals with πphase difference and four signals with $\frac{\pi }{2}$ phase difference were separately connected to signal synthesis unit. Figure 4b,c show the output signals. The integrated signal of the four coil’s signals (Figure 4c) is corresponding with output $V_{\mathrm{out}}$ in Figure 1f. Therefore, the circuits of the sensing system could achieve the designed function. However, the signals in Figure 4 were distorted, especially the waveform of signal (b) and (c). The use of diodes and relative high frequency excitation signal (1 MHz) might cause the phenomenon. The diodes would cause current overshoot. The high frequency excitation signal would induce the interference among the electronic components and wires. Both the current overshoot and the interference are harmful to the output signal. Especially when two or more signals are integrated into one output signal, the harmful components are overlapped together. The distortion output signal $V_{\mathrm{out}}$ would cause an unstable baseline of final voltage output to some extent.

### 3.2. Crosstalk Analysis

**Figure 5:**
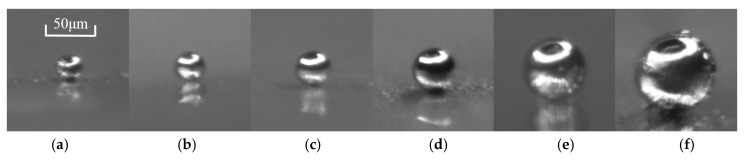
Iron particles used in pseudo-dynamic testing, (**a**) 29 μm, (**b**) 33 μm, (**c**) 38 μm, (**d**) 45 μm, (**e**) 66 μm, and (**f**) 83 μm.

**Figure 6:**
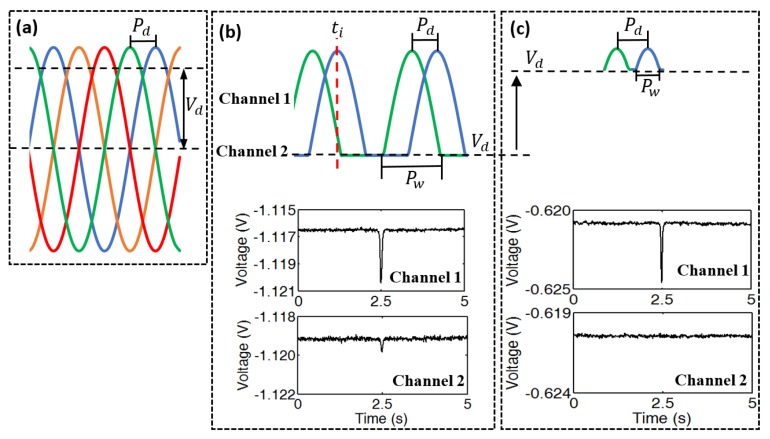
Illustration of crosstalk, (**a**) Signals at output port of four sensor units, (**b**) Crosstalk appears in channel 2 when 66 μm iron particle is passing through channel 1, (**c**) Voltage $V_{d}$ is increased to eliminate the crosstalk effect.

To conduct the crosstalk analysis, an iron particle (Φ 66 μm, as shown in Figure 5e) that was attached to the tip of nylon fiber (Φ 91 μm) with glue was used. When comparing to iron particles, the nylon fiber would cause negligible voltage change to the output [21]. The synchronized sampling and signal extraction method were used to monitor four sensing channels. When the iron particle was passing through the sensing coil of channel 1, a great pulse appeared in the output of channel 1. However, crosstalk also simultaneously appeared in channel 2. In order to find out the reason for crosstalk phenomenon, voltage $V_{d}$ was adjusted to check its influence for the procedure of signals synthesis. The results show that when voltage $V_{d}$ (horizontal black dotted line in Figure 6a) was decreased to the level shown in Figure 6b, crosstalk would appear. As shown in Figure 6b, decreasing the voltage $V_{d}$ would increase the signal waveform phase width $P_{w}$ at the diode output port that is larger than twice the phase difference $P_{d}$. Accordingly, when particle was passing though channel 1, impedance response would be overlapped in channel 2, shown at time point $t_{i}$ (red dotted line in Figure 6b), and then crosstalk appeared in the output of channel 2. If increasing the voltage $V_{d}$ level from Figure 6b to Figure 6c, the signal waveform phase width $P_{w}$ would decrease, and crosstalk would be eliminated, as shown in Figure 6c. Thus, in order to decrease the signal waveform phase width $P_{w}$ to smaller than twice of the phase difference $P_{d}$, the amplitude of input signal (A) and the voltage $V_{d}$ (as stated in Section 2.1) should the satisfy condition:

(6) $$A>V_{d}>A\sin\left(\frac{\pi }{2}-P_{d}\right)$$

From the condition (6), crosstalk would only appear when $V_{d}$ is smaller than $A\sin\left(\frac{\pi }{2}-P_{d}\right)$. Either reducing the input signal amplitude *A* or increasing the voltage $V_{d}$ at output port of diode could eliminate crosstalk. In the actual circuits, the signal waveform phase width $P_{w}$ is larger than its value in theory, and the initial phase change that is induced by large particle would change the phase difference $P_{d}$ between the adjacent signals. Both factors would render the condition (6) unable to be satisfied. Thus, lower input signal amplitude A or higher voltage $V_{d}$ is needed to guarantee that no crosstalk would appear in the final voltage output.

### 3.3. Pseudo-Dynamic Testing

**Figure 7:**
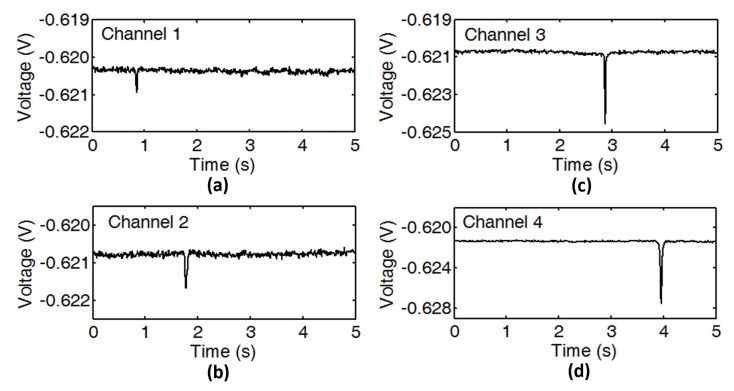
Measured voltage pulses caused by iron particles with the diameters of (**a**) 38 μm, (**b**) 45 μm, (**c**) 66 μm, and (**d**) 83 μm.

To validate the sensing mechanism, pseudo-dynamic testing was conducted while using iron particles. The sizes of iron particles are 38 μm, 45 μm, 66 μm, and 83 μm in diameter separately, as shown in Figure 5c–f. As the particles are almost spherical, the diameters could be directly measured from photo. Four particles, which were attached to the tip of nylon fiber, were driven by a stepping motor to have the same speed (2 cm/s). When four particles were dragged through the sensing coils at different times, particles’ corresponding pulses also showed at different times (Figure 7). The voltage change is increasing with the particle size, and no crosstalk is observed in the outputs. Even if two particles were passing through two sensing coils at the same time, two pulses would separately appear in two sensors’ outputs. This demonstrates that four sensing coils could independently conduct wear debris detection.

**Figure 8:**
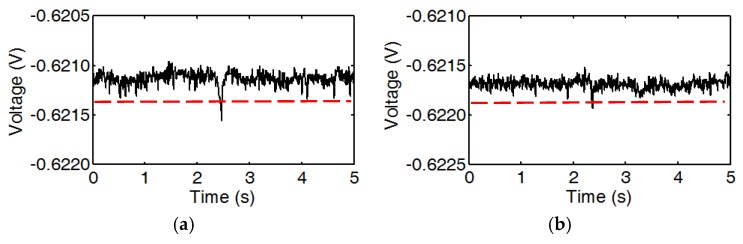
Measured voltage pulses caused by iron particles, (**a**) 33 μm, and (**b**) 29 μm.

Next, particles smaller than 38 μm were tested to determine the sensitivity of the sensor. The results show that particles as small as 33 μm (Figure 5b) could also be detected. The pulse could be obviously differentiated from the noise (Figure 8a). The signal-to-noise ratio (SNR) of the pulse is 1.22. Even through particles smaller than 33 μm (29 μm shown in Figure 5a) were tested, the response is almost negligible from background noise (Figure 8b). Thus, the detection limit of the sensor is 33 μm.

### 3.4. Dynamic Testing

Lubricating oil (Alexia S6, Shell Ltd., Hague, Dutch) mixed with iron particles was used to test the phase division multiplexed four-channel device. Iron particles, size ranging from 38 to 74 μm, were selected from iron power (Nangong Xindun alloy welding material spraying Co., Ltd., Nangong, China) by two stainless steel sieves (mesh 400 and mesh 200). 1.5 mg iron particles were mixed with 100 mL lubricating oil sample. The oil sample was loaded to pass through the sensing coils by a syringe pump. The flow rate was set to be 0.15 mL/min for each sensing channel. For the internal diameter of sensing coil being 400 μm, which is larger than particle size, no channel was clogged during the testing procedure.

**Figure 9:**
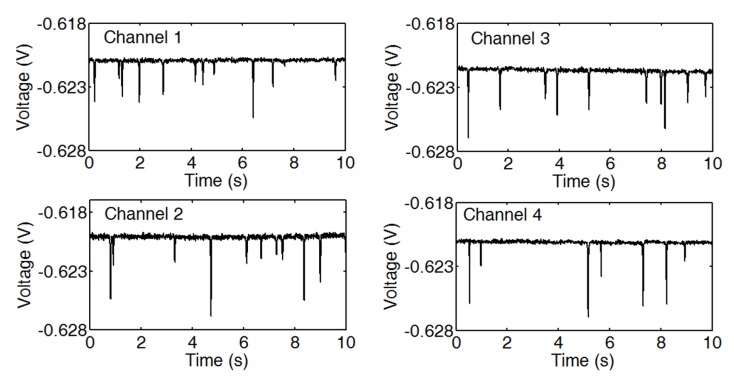
Measured voltage pulses for multichannel sensor with iron particles size ranging from 38 to 74 μm, and three-dimensional (3D) solenoid coil (390 μm in axial length, 400 μm in internal diameter, 1700 μm in external diameter, 65 μm in wire diameter).

Figure 9 shows the output signals of four channels and the supplementary video displays the continuous detection results. This test demonstrates that the device could simultaneously achieve real-time wear debris detection in the four channels by using the phase division multiplexing. In each channel, the pulse amplitude is corresponding to the size of particle passing through the sensing coil. As the four channels work independently, the throughput was increased to four times. With the same frequency of input signal and the same signal processing circuits, the detection sensitivities of four channels are comparable. When comparing with other multichannel wear debris sensors, only simple data sampling and signal extraction methods that were programed in LabVIEW are needed in this experiment. No additional post-processing program was used to calculate the final results. Thus, real time outputs of four sensing coils could be obtained.

### 3.5. The Expansibility of Phase Division Multiplexing

The proposed method also permits a larger number of channels to be installed in the sensing system. However, the crosstalk effect limits the increasing of the channel number. As stated in Section 3.2, only if the signal waveform phase width $P_{w}$ at diode output port smaller than twice of the phase difference $P_{d}$, crosstalk could be eliminated. For the phase difference, the number of channels N determine $P_{d}$.

(7) $$N\times P_{d}=2\pi$$

From conditions (6) in Section 3.2 and (7), we can get inequality:

(8) $$N<\frac{4\pi }{\pi -2\sin^{-1}\frac{V_{d}}{A}}$$

The number of channels N is limited by the proportion $\frac{V_{d}}{A}$. The higher the proportion $\frac{V_{d}}{A}$, the larger the number of channels N could be integrated into the multichannel sensing system. Decreasing the amplitude of input signal (A) or increasing the voltage $V_{d}$ in signal synthesis unit could obtain a higher proportion $\frac{V_{d}}{A}$. Correspondingly, the circuits of sensing system, sampling, and signal extraction program of the multichannel sensing system need to be adjusted for certain number of channels.

## 4. Conclusions

A multichannel wear debris sensor that is based on phase division multiplexing for oil condition monitoring was presented. The outputs of four sensing coils were obtained with only one sinusoidal excitation signal and one sampling channel. Synchronized sampling and signal extraction methods were used to record and recover the four channels’ output signals. Reducing the amplitude of input signal or promoting the voltage $V_{d}$ at the output ports of diodes eliminated the crosstalk effect. Dynamic testing was conducted with iron particles ranging in size from 38 to 74 μm to verify the sensing mechanism. The sensing coils are of the same sensitivity with the same excitation frequency and the same signal detection circuit. The simple data sampling and signal extraction methods that were used in the experiment largely decrease the data processing time. The proposed multichannel wear debris sensor could simultaneously detect wear debris in four channels without increasing the number of excitation source and data acquisition equipment. With the presented sensing mechanism, real time high throughput wear debris detection for lubrication oil condition monitoring could be achieved.

## Figures and Tables

**Table 1:** Parameters of two adjustable resistors $R_{\mathrm{adj}}$ in theory.

Phase Shift Unit#	Phase Shift Angle	$R_{adj}\;1\;(\Omega )$	$R_{adj}\;2\;(\Omega )$
Unit 1	0	+∞	+∞
Unit 2	$\frac{\pi }{2}$	$\frac{1}{\omega C}$	+∞
Unit 3	π	0	+∞
Unit 4	$\frac{3\pi }{2}$	0	$\frac{1}{\omega C}$

**Table 2:** Experimental parameters of the sensors and the corresponding capacitors.

Sensor#	$L_{s}\;(\mu H)$	$R_{s}\;(\Omega )$	$C_{p}\;(\mathrm{nF})$
Sensor 1	2.57	2.72	8.94
Sensor 2	2.54	2.68	9.04
Sensor 3	2.56	2.70	8.97
Sensor 4	2.59	2.75	8.87
